# The New York Institute of Stomatology

**Published:** 1904-06

**Authors:** 

**Affiliations:** The New York Institute of Stomatology


### Reports of Society Meetings.

### THE NEW YORK INSTITUTE OF STOMATOLOGY.

A meeting of the Institute was held at the "Chelsea," No. 222 West Twenty-third Street, New York, on Tuesday evening, February 2, 1904, the President, Dr. Brockway, in the chair.

Owing to the amount of material in the regular transactions, the communications on theory and practice were omitted.

The regular subject of the evening, Oral Hygiene and Prophylaxis, was discussed.

Dr. Sinclair Tousey stated that Dr. Leo Green and he had been working for some time on a method for the treatment of pyorrhœa alveolaris by means of the X-ray apparatus. They had quite a number of successful results to report later, but as yet they were not quite ready. Any members of the society who were interested he would like to invite to a demonstration of the apparatus, either at the St. Bartholomew's clinic or at his office. He also had a specimen of radium. He would also demonstrate a method he had perfected of the immediate development of the picture taken by means of the X-ray machine and without any dark room, only a comparatively dark space being necessary. The print could be ready for exhibition in five minutes after the exposure.

Upon motion, the thanks of the Institute were extended to Dr. Tousey for his kind offer.

Dr. E. S. Gaylord, of New Haven, stated that the subject for this evening, oral prophylaxis, was a most important and interesting one, yet he had not prepared a paper, and the little he had to say would be more in description of the work of another than anything he had been able to accomplish himself. In July last it was his good fortune to receive an invitation from Dr. D. D. Smith, of Philadelphia, to attend a clinical examination of a number of his patients' mouths who had been under his care for several years. There were eleven other dentists from different parts of the country in attendance. Dr. Smith had made appointments with his patients, to avoid confusion, and upon arrival, they were introduced, and a brief description given of the prior condition of the mouth by Dr. Smith, supplemented by the patient. Each gentleman was given the opportunity to thoroughly examine the mouth, Dr. Smith requesting not only a thorough examination, but to criticise whatever they might find. The first patient arrived soon after nine in the morning, the list of patients was completed a little after four, and they had examined twenty-nine mouths. Until about a dozen patients had been examined, it was his impression (and, he afterwards learned, that of others) that they were examining selected cases, and certainly those that required no dental operations, being in condition of perfect health. Their verdict was unanimous: positively there was no room for criticism, either of the fillings, crowns, or bridges, or general condition of the mouths; they seemed as nearly perfect as possible. In November last he again received an invitation from Dr. Smith, and with seventeen other dentists, representing nearly as many States, they spent nearly the entire day in examination of the mouths of thirty patients. Thus he has seen over fifty of Dr. Smith's patients, ranging from the ages of six to sixty-five. In many there were evidences that they had not been in a normal condition, and by the testimony of the patient, they learned there had been much suffering, both physical and mental, prior to Dr. Smith taking them in charge. Pyorrhœa in its worst form had been perfectly eliminated, and around the teeth was to be seen the beautiful restorations of the gingivæ, teeth firm in their sockets, entire surfaces beautifully polished, striations of the gums, giving evidence of a condition with which, in their own practice, they thought themselves unfamiliar; indeed, there was such sameness and perfect condition, that, as some one remarked, it seemed monotonous. In children's mouths they saw no evidence of caries having occurred, and he believes he is safe in affirmation that no caries occur in any of these mouths when well under treatment, and here it seems to him is where Dr. Smith is deserving of so much praise, in conceiving and executing the necessity of frequent treatments, which consist in systematically polishing the entire surfaces of the teeth every month with pumice and orange-wood stick, at the same time massaging the gum margins, thus not only preserving a condition of health, but seemingly a restoration or preservation of their natural colors; certain it is there was little variation in the shade of the teeth, even in different mouths. Dr. Smith asserts, and it seems reasonable, this rubbing with stick and pumice stimulates circulation within the tooth, thereby preserving natural hues and normal conditions. Dr. Smith has designed a set of scalers, both unique and unusual in form (made by J. W. Ivory), in that they are to be used in the manner of a file, with draw cut, which after removal of the calculus, leaves the surface of the teeth smoother than the ordinary scaler, and able to follow into pockets farther with greater ease and less pain than with any other form with which he was familiar. After thorough scaling, the teeth are thoroughly polished with the orange-wood and pumice, held in a very neat form of porte-polisher of his own device, and each tooth is polished, not only on its crown, but, where pyorrhœa exists, to the bottom of each pocket; the pockets are then washed with a strong solution of phenol sodique, after which, in severe cases, a deliquesced solution of zinc chloride is carried in with the point of a small stick, the ordinary wood tooth-pick shaved thin serving a good purpose. Dr. Gaylord regards the opportunity of seeing this method of treatment of more value than he can well express, and he has carried it into his own practice with results that are so pleasing to his patients and himself that he will never abandon it. He would urge all to investigate, particularly the young men, as he believes it possible to conduct a practice, beginning with children, carrying them along through life with no fillings (except in imperfections in fissures), without the loss of a single tooth, and pyorrhœa absolutely unknown to them.

Dr. C. W. Strang was reminded of what Dr. Dio Lewis had said many years ago, that a clean tooth never would decay. If Dr. Lewis had stated that a perfectly formed tooth, properly kept clean, would never decay, Dr. Strang would feel more like agreeing with him. For that thing to be accomplished, the patient must polish his teeth almost every minute of the day. Dr. Strang mentioned the case of an old patient of thirty years' standing in his practice who from year to year had had very little to be done with the exception of the removal of a little soft tartar and the polishing of the teeth. Occasionally there had been a little filling to be inserted. About two years ago that gentleman's health had failed, and six months ago Dr. Strang had seen the patient and noted evidences of a change in the condition of his mouth. At that time he had been entered upon his books to be seen again in six months. At the end of this six months he was surprised to see the ravages of disease in that mouth. Dr. Strang believed that such progress of dental caries as this was due principally to the vitiated condition of the secretions of the mouth, more than to deteriorations of tooth-structure. Dr. Black has told us that there is very little difference in the various specimens of dentine that he had examined. In the enamel there is a great difference. About five years ago Dr. Strang had made the statement before a local society in Providence that if we took patients in early life and were systematic and careful in cleansing and polishing their teeth we would have very little trouble from the development of pyorrhœa alveolaris in these cases. Dr. Strang had been fortunate enough when in college to be under the tuition of Dr. James Truman. Dr. Truman had always tried to impress upon the minds of the students the importance of keeping the teeth of every patient clean; that the younger men could not hope to compete with the older practitioners in many things, but they could compete in cleanliness. Dr. Strang had always endeavored to follow this teaching to his very best ability. At first the instruments obtainable for this purpose were very crude, but with time these had improved. He had been conscientiously trying for the past thirty-five years as far as possible to remove the deposits of tartar from his patients' teeth, but he did not believe he was any better able to do it to-day than he was five years ago, except that now he was equipped with a little better instruments. If anybody had told him two years ago that he was not doing as well by his patients as he ought, he would not have believed them; indeed, he would have been willing to compare any twenty-five or thirty of his patients with those of any other practitioner. He was glad that Dr. D. D. Smith had not had an opportunity to accept such a challenge. If any one had told him two years ago that teeth could not be properly cleansed with the dental engine, Dr. Strang would have said that he knew better. When he had seen the results of the work in Dr. D. D. Smith's office, it did not take him long to be convinced that he had been doing the work improperly. He had been so thoroughly convinced that since last July he had used the dental engine but three times in cleansing teeth, and then only to remove some stains.

Dr. Alfred C. Fones stated that he claimed little or no originality regarding the thoughts he wished to express on this subject. Our chief object should be the prevention of dental diseases, rather than being content with the repairing of the damages wrought. He said that micro-organisms, with their action upon food débris, were the cause of most of the pathological conditions found in the mouth. As it was seemingly impossible to entirely rid the mouth of these organisms, we should adopt some plan or system whereby our patients could keep their mouths clean and free from the accumulations of food products. The teeth presented about twenty-five square inches of surface. Now, if we compared the relative difficulties of cleansing a piece of smooth and then of ground glass of about this surface, the importance of keeping the teeth smooth and polished could readily be seen. The method used to produce this mirror surface was that advocated by Dr. D. D. Smith, using pumice and orange-wood sticks carried in holders, such as furnished by J. W. Ivory of Philadelphia. All surfaces of the teeth are thoroughly rubbed, considerable pressure being used, the edge of the stick kept sharp, in order to reach under the margin of the gums. Particular attention is paid to the necks of the teeth. Polishing strips are used on the proximal surfaces. It takes, generally, a number of thorough treatments to produce this high polish, but when once secured it may easily be maintained by monthly treatments of about a half-hour.

He said it was absolutely impossible to get the desired results unless we had the thorough co-operation of our patients and were arbitrary with them in insisting upon their systematically cleansing their teeth with tooth-brush and dentifrice. We should also prescribe the kind of brush and kind of dentifrice, and when and for how long they should cleanse their teeth. If we would be arbitrary in these matters it would be done as we directed and we would soon begin to get results. The average person spends, perhaps, twenty to thirty seconds brushing the teeth, in spite of what we all have heard them affirm,—that they spent fully five minutes at it,—evidently never having timed themselves. It was impossible to get proper results in less than ninety seconds for thoroughly cleansing the teeth and imparting a vascular stimulus to the gums.

Dr. Fones stated that he had some forty patients under practical treatment in this manner, and was much pleased with the results obtained. Through Dr. Smith he had become somewhat interested in the system some five years ago, but had not then realized its full value. He believed it was along the line of prophylaxis that future progress in the profession of dentistry would be made. He believed that the art of restoring lost tooth-structure had about reached its zenith, unless perhaps the ideal filling, a porcelain cement, might be discovered. The work of the future in preventing dental caries and the development of pyorrhœa would be found in extreme cleanliness. He said it was an ideal treatment for children's teeth and for adults who were susceptible to dental decay. After a thorough instrumentation in cases of pyorrhœa, he believed it to be the most effective treatment known.

Regarding the theoretical side of pyorrhœa alveolaris, Dr. Fones considered the disease divided into (1) interstitial, (2) phagadenic pericementitis, and (3) gouty pericementitis, the third being rare, the first and second being conditions we were constantly meeting. It had been estimated that sixty-five per cent. of the teeth lost were lost from results of this disease. Although caries was more prevalent, it was not so fatal. He thought that the development of the disease might be placed under two general heads, one due to susceptibility and the other to a local exciting cause.

Every person, after passing the age of thirty-five, was susceptible to this disease; it was simply a question of degree. After this age the peridental membrane gradually became thinner, the alveolar process more dense, and there was a lessened vascularity and resistant power. Susceptibility of the patient was increased by nervous disorders, lowering the vitality, faulty metabolism, etc. The exciting cause was almost entirely produced by the irritation of the toxins and ptomaines generated by the action of micro-organisms on food débris around the necks of the teeth. Its quick response to extreme cleanliness and antiseptic treatment seemed to prove it. The stimulus imparted to the gums by the thorough and systematic brushing was imparted in turn to the underlying tissues, which lessened their susceptibility by increasing their vascularity.

To what degree these nervous disorders and faulty metabolic conditions were caused by systematic infection due to unsanitary mouths, it was difficult, at present, to predict. Chronic indigestion, neurasthenia, malarial symptoms, periodic headaches, throat troubles, and bad breath were cured or showed marked improvement in a comparatively short time. He believed there was a condition here producing systemic disturbances that the great majority of the medical profession had not dreamed of. The time was coming when the first law of hygiene would be recognized as a clean and sanitary mouth, and the assistance of the dental practitioner would prove indispensable to his medical brother in restoring many of his patients to health without the use of drugs.

Dr. Fones believed that the dental profession will owe Dr. D. D. Smith a debt of gratitude for demonstrating and calling our attention to this field of prophylaxis which is now opening up before us.

Dr. L. C. Taylor merely wished to reinforce the statements made. He thought the subject of Oral Hygiene and Prophylaxis a very important one. A vast number of people are to-day suffering directly or indirectly from mouth infection, and many cases of so-called stomach trouble can be cured by proper attention to oral cleanliness. It requires great skill to treat a set of teeth properly where pyorrhœa alveolaris is present. He mentioned a friend who thought a higher grade of skill is necessary than in an operation for appendicitis.

Several cases were mentioned of invalids being entirely cured by a careful attention to the septic condition of the mouth and polishing off the bacterial plague on the teeth. They had been suffering from mouth infection and nothing more. We have had ample description and abundance of literature upon the different kinds of bacteria found in the oral cavity. Dr. Andrews, Dr. Williams, Dr. Black, and may others have given us the result of their researches, but none have told us how to get rid of this infection. It is time that we get to the business side of it. We know these bacteria existed, we have seen them repeatedly, but have we seen the beautiful healthy mouth and the resulting tone of the whole system? This is what we want. It is a high ideal, but it is one worth attaining.

Regarding the much talked of connection between uræmia and pyorrhœa, and the consequent desirability of a systemic treatment for pyorrhœa, Dr. Taylor did not know of a single instance where a systemic treatment had cured a case of pyorrhœa, but he could mention numbers of cases where, the pyorrhœa alveolaris being cured, the uræmia had disappeared. It was another case of intestinal derangement due to mouth infection.

Dr. Taylor does not believe any living man can properly treat pyorrhœa of the mouth with the dental engine. He mentioned in this connection that the first practical teaching of oral hygiene and prophylaxis in any dental college was established in a dental school in New York City. He said he thought New York would see the time when she would be proud of this pre-eminence.

Dr. Taylor spoke with praise of the work done by Dr. D. D. Smith, of Philadelphia. He thoroughly approves his methods. He does not believe that in many cases it is possible at one sitting to get perfect health of the mouth. It will require from twelve to twenty consecutive months before that mouth will be satisfactory to the practitioner. The first two or three sittings will be sufficient to render the teeth mechanically clean, and thereafter each month the teeth may be gone over in thirty or forty minutes, but it will take months for the mouth to reach what might be called a perfect state of health.

Dr. Tousey stated that in the work at the St. Bartholomew's Parish, it was noticed that an unhygienic condition of the patient's mouths existed almost without exception, and in order to stimulate the children to be more cleanly they were taught to play what was known as the "tooth-brush game." The game had become very popular, and it was seen that some of the elders and parents were playing the game also.

Dr. Green stated that Dr. Fones's remarks about the care of children's mouths had led him to call attention to other features in this connection. He believed that prophylaxis should be extended to include proper nourishment even before the eruption of the teeth. He had noticed, especially among the children of wealth, that the modified forms of milk so commonly given to children had a very deleterious effect upon their teeth. These foods seemed to lack the essential properties required in tooth building. Furthermore, Dr. Green thought, where children are given over to a wet-nurse, too much attention could not be given to the condition of the mouth of the nurse. Dr. Green had been preparing statistics upon this subject, but as yet was not prepared to place them in the hands of the society.

Dr. Locherty stated that he would like to hear more concerning the medicines used in connection with this mechanical cleansing. He had himself, in the case of very persistent green stain, applied the rubber dam and first used iodine, following this with hydrogen peroxide in connection with a heated instrument, thus bleaching the surface and afterwards polishing. The results in these instances had been very satisfactory.

Dr. F. Milton Smith stated that in regard to this subject and the claim of Dr. D. D. Smith, of Philadelphia, it seemed to him that this perfect cleanliness of the teeth and mouth was the goal after which every man here was striving. That Dr. Smith succeeded better than any other man he knew he was willing to admit. He had seen a dozen or fifteen of these cases, and he hoped the time would come when he would be able to do as well by his own patients. However, he did not believe Dr. Smith was the first man to preach that cleanliness was the best thing we could accomplish for our patients. If he had understood Dr. Gaylord, Dr. D. D. Smith claimed nothing new except persistence. He was very glad to hear this. He had made a statement himself to the effect that he believed the chief point in Dr. Smith's method was thoroughness, which is another word for persistence. However, according to his own understanding of the case, this was not all that Dr. D. D. Smith had claimed for his method. He had claimed something entirely new and something that he alone had advanced. There seemed to be some mystery about this point. He himself believed and it was generally conceded that a thorough polishing of the teeth, even as often as once in two weeks or once a month was a very beneficial thing, and he had several cases where he had proved the efficacy of this. Dr. Gaylord had stated that he formerly believed the teeth could be thoroughly cleansed with the engine. Dr. Smith had never believed that. However, he believed that with the dental engine, with scalers, files, and scrapers, with the polishing sticks and pumice, with the floss-silk, and with every other means available, it would be possible to pretty nearly remove all the foreign substances from the teeth, and when this was done as thoroughly and as frequently as it was done by Dr. D. D. Smith the results would be marvellous.

The President stated that he was most favorably impressed with the prophylactic method under consideration, and had read all the papers describing it, published by Dr. D. D. Smith, with great interest and profit. As he had remarked on a former occasion, he was an easy convert to the idea, for the first operation at which he was set by his preceptor at the beginning of his study of dentistry was the cleansing and polishing of teeth in the mouths of his patients. His preceptor had been a strong believer in the value of such service, and Dr. Brockway had always retained and practised what he was then taught.

He thought, however, that few if any of us had carried the cleansing process to the extent and thoroughness of Dr. Smith, which is its most important and distinguishing feature, and for this Dr. Smith has rendered our profession and the public which we serve what Dr. Brockway considers the greatest service of our time.

At first he was inclined, as were others, to take issue with Dr. Smith on his emphatic declaration that this work could not be accomplished by the engine, and while Dr. Brockway still holds that much of it can more easily be done thereby, it is perhaps necessary to supplement its use by hand-work with the porte-polisher and similar adjuncts for the purpose of obtaining the stimulating effect aimed at.

In this connection he wished to call attention to the value of tecum fibre as a most efficient article for the purpose of polishing the approximal surfaces of the teeth where the floss-silk and tape are generally used, this being fine, strong, and charged with native grit, silex, like the cortex of a reed, making it most admirable for the purpose required. It can be drawn between the most closely set teeth with the aid of a little vaseline, in extreme cases, which does not seem to impair its efficiency. Dr. Brockway thought that probably this article was not new to most of the men present, but, never having seen any mention of it in the discussions of this subject, he could not refrain from calling attention to its merits.

A vote of thanks was extended to the gentlemen who had so kindly presented this subject to the Institute.

Adjourned.

Fred. L. Bogue, M.D., D.D.S.,
*Editor The New York Institute of Stomatology.*

